# *Leptomonas seymouri* Co-infection in Cutaneous Leishmaniasis Cases Caused by *Leishmania donovani* From Himachal Pradesh, India

**DOI:** 10.3389/fcimb.2020.00345

**Published:** 2020-07-15

**Authors:** Lovlesh Thakur, Hemant Ritturaj Kushwaha, Ajeet Negi, Aklank Jain, Manju Jain

**Affiliations:** ^1^Department of Zoology, Central University of Punjab, Bathinda, India; ^2^School of Biotechnology, Jawaharlal Nehru University, New Delhi, India; ^3^Department of Dermatology, Indira Gandhi Medical College, Shimla, India; ^4^Department of Biochemistry, Central University of Punjab, Bathinda, India

**Keywords:** *Leptomonas seymouri*, cutaneous leishmaniasis, *Leishmania donovani*, Himachal Pradesh, India

## Abstract

Himachal Pradesh in India is a newer endemic state with co-existence of cutaneous and visceral leishmaniasis. The cutaneous leishmaniasis cases are on an increase in the region and reported to be unusually caused by *Leishmania donovani* with limited molecular validation. In order to molecularly characterize the causative parasite of the cutaneous disease, parasite specific Internal-Transcribed Spacer 1 (ITS1) PCR RFLP and sequence analysis was performed on skin lesional biopsies from cutaneous leishmaniasis patients. Interestingly, we found the presence of *Leptomonas seymouri* in 38.5% (22/57) of the patients along with *L. donovani* detected in all the samples. *L. seymouri* is a monoxenous insect trypanosomatid, generally incapable of infecting humans. In recent years, the parasite is also reported to co-infect humans with *L. donovani* in visceral and post kala-azar dermal leishmaniasis (PKDL) cases prevalent in northeastern India. The finding of *L. seymouri*-*L. donovani* co-infection in unusual cutaneous cases from Himachal Pradesh is the first ever to our knowledge and imply a newer disease paradigm. There is an urgent need to understand the biology of *Leptomonas* co-infection with *L. donovani* and its possible role in visceral and/or dermotropic disease outcome. Importantly, *L. seymouri* co-infection in cutaneous cases and previously reported visceral and PKDL cases needs to be recognized as a newer phenomenon by the leishmaniasis surveillance program in India.

## Introduction

Leishmaniasis is a disease complex caused by *Leishmania* parasite with a digenetic life cycle in the sandfly vector and the mammalian host. In India, visceral leishmaniasis (VL) caused by *L. donovani* predominates in the northeast belt in the state of Bihar, West Bengal, Uttar Pradesh, and Jharkhand with fewer cutaneous leishmaniasis (CL) cases by *L. tropica* in the hot arid western region of the Thar Desert in Rajasthan (Thakur et al., 2018). More recently, newer endemic pockets in the state of Kerala and Himachal Pradesh (HP) in India are exhibiting unusual disease presentation with *L. donovani* causing cutaneous leishmaniasis (Sharma et al., 2005; Kumar et al., 2015; Thakur et al., 2018). In recent years, the hilly state of Himachal Pradesh in India is coming up with increased number of CL cases caused by *L. donovani* in the previously non-endemic zones (Sharma et al., 2005; Kumari et al., 2017). Lack of in-depth study on the involvement of *L. donovani* in CL cases from HP made us perform a comprehensive molecular analysis of the parasite in the patients' skin lesional specimens. In our study, we report for the first time the presence of *L. seymouri* co-infection in the unusual CL cases in Himachal Pradesh (HP) caused by *L. donovani* variants (unpublished data). Classically, *Leptomonas* spp. comprise insect parasite with a monoxenous life cycle and are considered non-pathogenic to humans. Lately, adaption of the parasite to dixenous life cycle has been reported under specific conditions such that *L. seymouri* can exist as a co-infectant with other pathogens in immunocompromised subjects (Dedet and Pratlong, 2000; Kraeva et al., 2015; Selvapandiyan et al., 2015; Kaufer et al., 2017). In this context, our finding re-affirms the limited but significant emerging evidence on the newer parasitic capability of *Leptomonas* sp. as an opportunistic human pathogen in CL cases from HP. This is in line with the previous reports on *L. seymouri* as a co-infectant in clinical isolates as well as direct clinical specimens from VL and PKDL cases from northeast India (Srivastava et al., 2010; Ghosh et al., 2012; Singh et al., 2013).

## Materials and Methods

### Study Design and Ethics

Sixty CL patients, indigenous to Sutluj river belt in Himachal Pradesh were included in the study over the period from 2014 to 2018. Lesional skin biopsies were collected from the patients at Department of Dermatology, Indira Gandhi Medical College (IGMC), Shimla and Mahatma Gandhi Medical Services Complex (MGMSC) Khaneri, Rampur, Shimla at the time of diagnosis. Written informed consent was obtained from all the patients. The study design was approved by the Institutional Ethics Committee IGMC, Shimla, Himachal Pradesh, Approval no. HFW(MS)G-5(Ethics)/2014-10886 and Central University of Punjab, Approval no. CUPB/IEC/2016/034.

### Clinical Confirmation of CL Patients: Parasite Detection and Histopathological Analysis

Lesional biopsy samples were processed for parasite detection using Giemsa stained touch smears, Hematoxylin and Eosin (H & E) stained paraffin-embedded tissue sections as per standard protocol (Elder et al., 2001; Bain et al., 2016). A part of the lesional biopsy from the CL cases was processed for examining CL specific histopathological changes. The samples were processed in 10% NBF, embedded in paraffin and processed to 4–5 μm thick tissue section. Tissue sections were stained with H&E and examined for histopathological changes specific to cutaneous lesions.

### Molecular Analysis: ITS1 PCR-RFLP

Molecular identification of the parasite was performed using skin lesional specimens from the clinically confirmed CL patients. Genomic DNA (gDNA) isolation from patient samples and laboratory-grown L. donovani (Ld1S2D, LdBob) promastigote culture was done using standard protocol (Salotra et al., 2001). gDNA from 57 samples were used for species-specific ribosomal Internal-Transcribed Spacer 1 (ITS1) region PCR RFLP assay as described previously (El Tai et al., 2000). ITS1 specific PCR amplification was done with the primer set LITSR (5′-CTGGATCATTTTCCGATG-3′) and L5.8S (5′-TGATACCACTTATCGCACTT-3′). Briefly 50–100 ng of gDNA was used as template and amplified with 10 pmol of each primer using Go Taq Green Master mix, 1X (Promega, Cat # M7122) with an initial denaturation at 95°C for 2 min, 34 cycles of denaturation at 95°C for 20 s, annealing at 53°C for 30 s and extension at 72°C for 1 min with the final extension at 72°C for 6 min. The PCR product of ~ 320 bp size was subjected to HaeIII RFLP with overnight HaeIII digestion at 37°C and run on 2.5% agarose gel. The amplification product of ~320 bp and ~400 bp were eluted using Qiagen kit and submitted for Sanger sequencing for parasite identification.

### Sequence Alignment and Phylogenetic Analysis

Sequences corresponding to the ~320 bp and ~400 bp amplification products were retrieved for the representative samples with their accession numbers deposited in Genbank (Table 1). Each of the sequences was analyzed using BLAST with default parameters to identify the parasite in the cutaneous lesions. ITS1 nucleotide query sequences were aligned with relevant ITS1 reference sequences of standard WHO and region specific *Leishmania* spp., *Leptomonas* spp. and *Trypanosoma* spp. isolates using multiple alignment software, MUSCLE using default parameters (Table 1) (Edgar, 2004). The sequence alignment was done using Jalview multiple alignment editor version 2.10.4b1 (Clamp et al., 2004). The maximum likelihood tree from the aligned sequences was obtained with 1000 bootstraps with default parameters using the *dnaml* program of the phylip package (Felsenstein, 2004). The final tree was plotted using FigTree software (version 1.4.3).

**Table 1:** GenBank Accession Numbers of ITS1 sequences of standard *Leishmania, Leptomonas*, and *Trypanosoma spp*. and ITS1 test sequences from CL patients used in phylogenetic analysis.

**Species**	**WHO code**	**GenBank Accession Number**
***Leishmania spp***.		
*L. donovani* (India)	MHOM/IN/00/DEVI	AJ634376
*L. donovani* (Sri Lanka)	MHOM/LK/2002/L60c	AM901447
*L. donovani* (Bhutan)	Not Available	JQ730001
*L. major*	MHOM/SU/73/5ASKH	AJ000310
*L. tropica* (India)	MHOM/SU/60/OD	EU326226
*L. mexicana*	MHOM/MX/85/SOLIS	AJ000313
*L. amazonensis*	MHOM/BR/73M2269	HG512964
***Leptomonas spp***.		
*L. seymouri* (India)	ATCC 30220	EU623433
*L. seymouri* (India)	Not Available	JN848802
*L. seymouri* (India)	Not Available	KP717899
***Trypanosoma spp***.		
*T. brucei*	Not Available	X05682
*T. cruzi*	Not Available	L22334
**Accession no. of ITS1 sequences**, **~320 bp from samples showing single band in ITS1 PCR**		
HPCL49 Ld		MG982978
HPCL52 Ld		MG982981
**Accession no. of ITS1 sequences**, **~320 bp from samples showing dual bands in ITS1 PCR**		
HPCL4 Ld		MG982942
HPCL17 Ld		MG982951
HPCL34 Ld		MG982965
HPCL36 Ld		MG982967
HPCL38 Ld		MG982969
HPCL39 Ld		MG982970
HPCL41 Ld		MG982971
HPCL42 Ld		MG982972
HPCL43 Ld		MG982973
**Accession no. of ITS1 sequences**, **~400 bp from samples showing dual bands in ITS1 PCR**		
HPCL4 Ls		MH537621
HPCL17 Ls		MH537622
HPCL34 Ls		MH537623
HPCL36 Ls		MH537624
HPCL38 Ls		MH537625
HPCL39 Ls		MH537626
HPCL41 Ls		MH537627
HPCL42 Ls		MH537628
HPCL43 Ls		MH537629

*Country of origin of the standard strain is written in parentheses*.

## Results

### Clinical and Histopathological Confirmation of the CL Patients

In the present study, 60 patients with typical cutaneous lesions presented as plaques, nodules and/or papules were included. Most of the patients exhibited typical localized cutaneous lesions, often with raised borders, serous crusting and ulceration. The lesion number and size range in males and females is given in Supplementary Table 1 separately for *L. donovani*-*L. seymouri* co-infection cases and *L. donovani* alone infected cases with no major difference in the two groups. It is important to mention that all the CL cases analyzed were indigenous in nature with no VL history or any visit to VL endemic area. In both *Leptomonas* co-infected cases and *L. donovani* alone infected CL cases, males and females were almost equally represented with maximum disease frequency in the age group of 21–40 years, summarized in Supplementary Table 1.

All the patients with characteristic CL lesions were clinically confirmed for the presence of amastigotes in Giemsa stained lesional touch smears and Haematoxylin/Eosin stained paraffin embedded tissue sections (Supplementary Figures 1A,B). In Giemsa stained tissue smears, 35% of the samples were amastigote positive from the *L. donovani*-*L. seymouri* co-infected cases while ~65% samples were positive for amastogotes in *L. donovani* alone infected cases. Interestingly, ~ 40% of the Haematoxylin/Eosin stained biopsy samples were parasite positive for both the groups (Supplementary Table 1).

The CL lesion-specific histopathological analysis of biopsy samples from both the co-infected and *L. donovani* alone infected cases, showed characteristic CL lesion-specific epidermal changes with acanthosis and papillomatosis along with granulomatous inflammation (Supplementary Figures 2A,B). No apparent differences in the histopathological features were observed in the two groups with level of histopathological manifestations varying from sample to sample.

### ITS1 PCR RFLP Based Identification of the Parasite in the CL Patients

**Figure 1:**
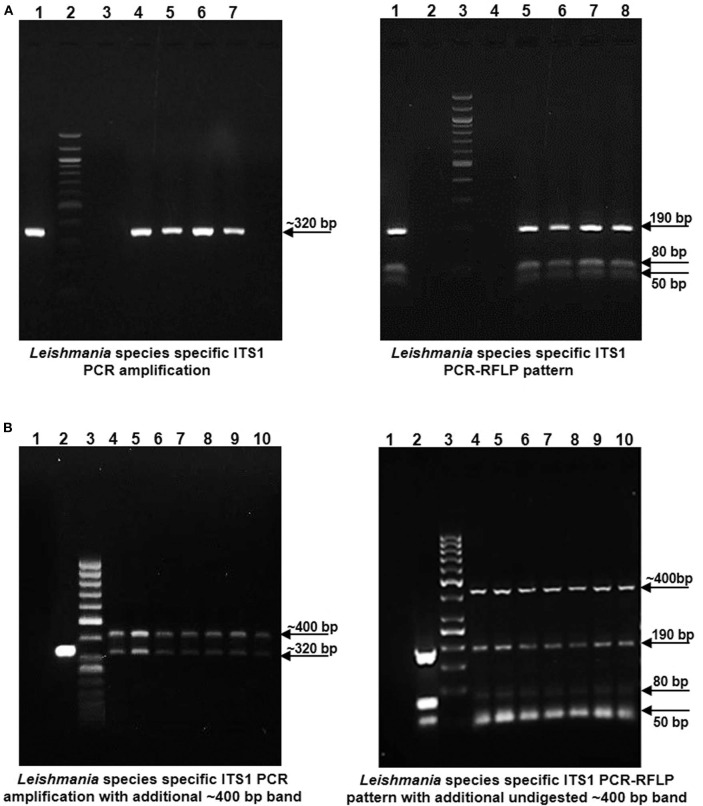
*Leishmania* species specific ITS1 PCR on DNA isolated from lesion biopsy samples from CL patients and HaeIII PCR RFLP analysis of ITS1 region of test samples and *L. donovani* culture as a positive control. **(A)** Left Panel: Representative patient samples with a single, *Leishmania* spp specific ~ 320 bp amplification product. Lane 1, *L. donovani*; Lane 2, 100 bp DNA marker; Lane 3, water control; Lanes 4–7, CL test samples. Right Panel: HaeIII ITS1 PCR-RFLP pattern for identification of *Leishmania* species. Lane 1, *L. donovani*; Lane 3, 100 bp marker; Lane 4, water control; Lanes 5–8, CL test samples. **(B)** Left Panel: Representative patient samples with dual amplification bands (~ 320 bp and ~ 400 bp). Lane 1; Water control, Lane 2; *L. donovani* positive control, Lane 3; 50 bp DNA marker, Lanes 4–10, CL test samples. Right Panel: HaeIII ITS1 PCR-RFLP pattern of samples with dual bands. Lane 1; Water control, Lane 2; *L. donovani* positive control, Lane 3; 50 bp DNA marker, Lanes 4–10, CL test samples.

gDNA samples from lesional skin biopsies of 57 CL patients were used for parasite species specific identification by PCR RFLP analysis of ITS1 region along with gDNA from standard *L. donovani* (1S2D, LdBob) culture used as a positive control. HaeIII restriction enzyme digestion of the amplified ITS1 region between the small subunit (ssu) rRNA and 5.8S rRNA DNA provides a *Leishmania* spp. specific RFLP pattern (Dávila and Momen, 2000; El Tai et al., 2000). ITS1 specific PCR amplification gave the expected ~320 bp product in all the 57 CL samples similar to the positive control while 38.5 % (22/57) samples gave a dual band pattern with a unique ~400 bp extra band (Figures 1A,B, Left Panels). The ~320 bp band in all the samples resolved into HaeIII RFLP pattern identical to the *L. donovani* control sample with 3 discrete fragments of ~190 bp, ~80 bp, and ~50 bp (Figures 1A,B, Right Panels). The extra ~400 bp band, specific to 22 samples remained undigested as depicted in Figure 1B, Right Panel. Our result suggests possible co-infection of non-*Leishmania* trypanosomatid along with *L. donovani* in the test samples, based on existing literature. Ghosh *et al* reported a similar ITS1 PCR RFLP pattern in 4/29 of VL and 2/7 of PKDL specimens with the extra ~400 bp band corresponding to *L. seymouri* (Ghosh et al., 2012).

### Detection of *Leptomonas* Co-infection With *L. donovani* in the CL Patients

Sequence based identification of the ~320 bp and ~400 bp amplicons was performed for the representative CL samples (2 samples with ~320 bp band alone and 9 samples with dual band pattern). All the sequences corresponding to ~320 bp band and ~400 bp band were deposited with their accession numbers in Genbank (Table 1). ITS1 sequences corresponding to ~320 bp suggested all the samples to be closest with *L. donovani* with maximum identity to *L. donovani* isolate from Bhutan (JQ730001) using BLAST analysis while the sequences corresponding to ~400 bp band, showed maximum identity with the standard *L. seymouri* ITS1 sequences with accession numbers KP717899, EU623433, and JN84880 (Table 1) (Ghosh et al., 2012; Yangzom et al., 2012; and unpublished data). A similar finding with the unique ~400 bp band representing *L. seymouri* specific ITS1 sequence and ~320 bp band specific to *L. donovani* ITS1 sequence has been demonstrated earlier in the VL and/or PKDL patients (Ghosh et al., 2012).

**Figure 2:**
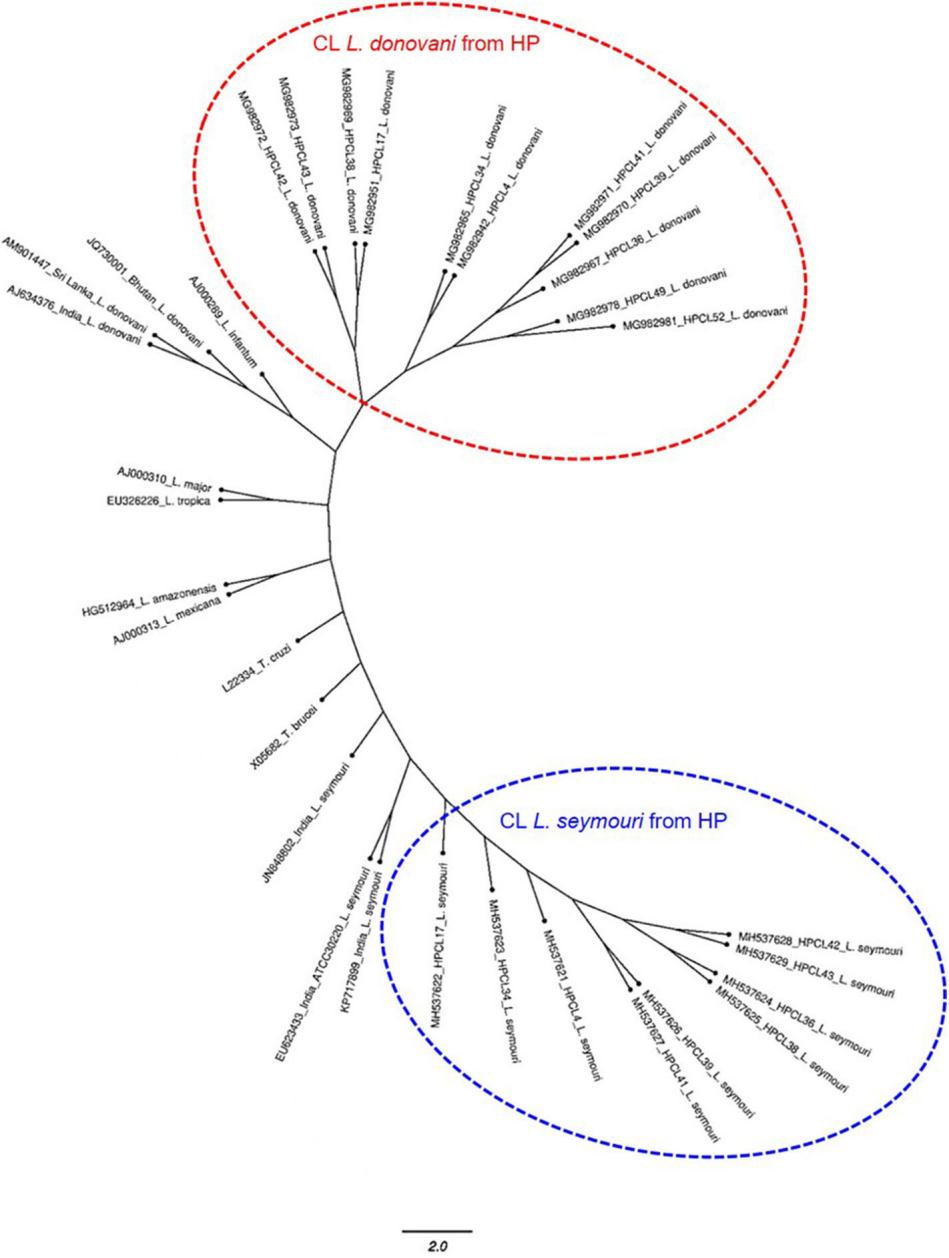
Phylogenetic tree of ITS1 gene sequences from CL test samples with single and dual-band amplification patterns (~320 bp and ~400 bp band size test samples, designated as HPCL_Ld series corresponding to ~320 bp band and HPCL_Ls series corresponding to ~400 bp band, numbered in order of their collection) and ITS1 sequences of standard *Leishmania, Leptomonas* and *Trypanosoma* isolates from Genbank was obtained using Maximum Likelihood method with 1,000 bootstraps using dnaml program of PHYLIP package.

The multiple sequence alignment of the test sequences (~320 bp and ~400 bp) from the CL samples and the ITS1 sequences representing standard *Leishmania, Leptomonas* and *Trypanosoma* isolates from GenBank was performed and used for phylogenetic analysis using maximum likelihood method (Table 1). Phylogenetic analysis grouped all the HPCL isolates into two discrete clusters corresponding to *Leishmania* and *Leptomonas* with respect to the ~320 bp and ~400 bp ITS1 sequences respectively (Figure 2). All the 11 CL test samples clustered closely with the standard *L. donovani* isolates from India, Sri Lanka and Bhutan and *L. infantum* with respect to the ITS1 specific ~320 bp sequences represented by HPCL_*L. donovani* series (Figure 2, Table 1). Sequence analysis using BLAST and phylogenetic classification, suggest that the CL in HP is caused by *L. donovani* isolates closest to the Bhutan *L. donovani* isolate and distinct from the Indian and Sri Lankan *L. donovani* isolates (unpublished data). Nine out of 11 CL test samples also clustered closely with the standard *L. seymouri* isolates from India with respect to the ~400 bp sequence represented by HPCL_*L. seymouri* series (Figure 2, Table 1). Interestingly, both the HPCL derived parasite clusters representing ITS1 sequences corresponding to *L. donovani* and *L. seymouri* exhibited considerable heterogeneity signifying sequence variation among multiple isolates analyzed. The standard isolates representing *Leishmania* spp. known to cause CL in old and new world and *Trypanosoma* spp clustered independently as outgroups.

**Figure 3:**
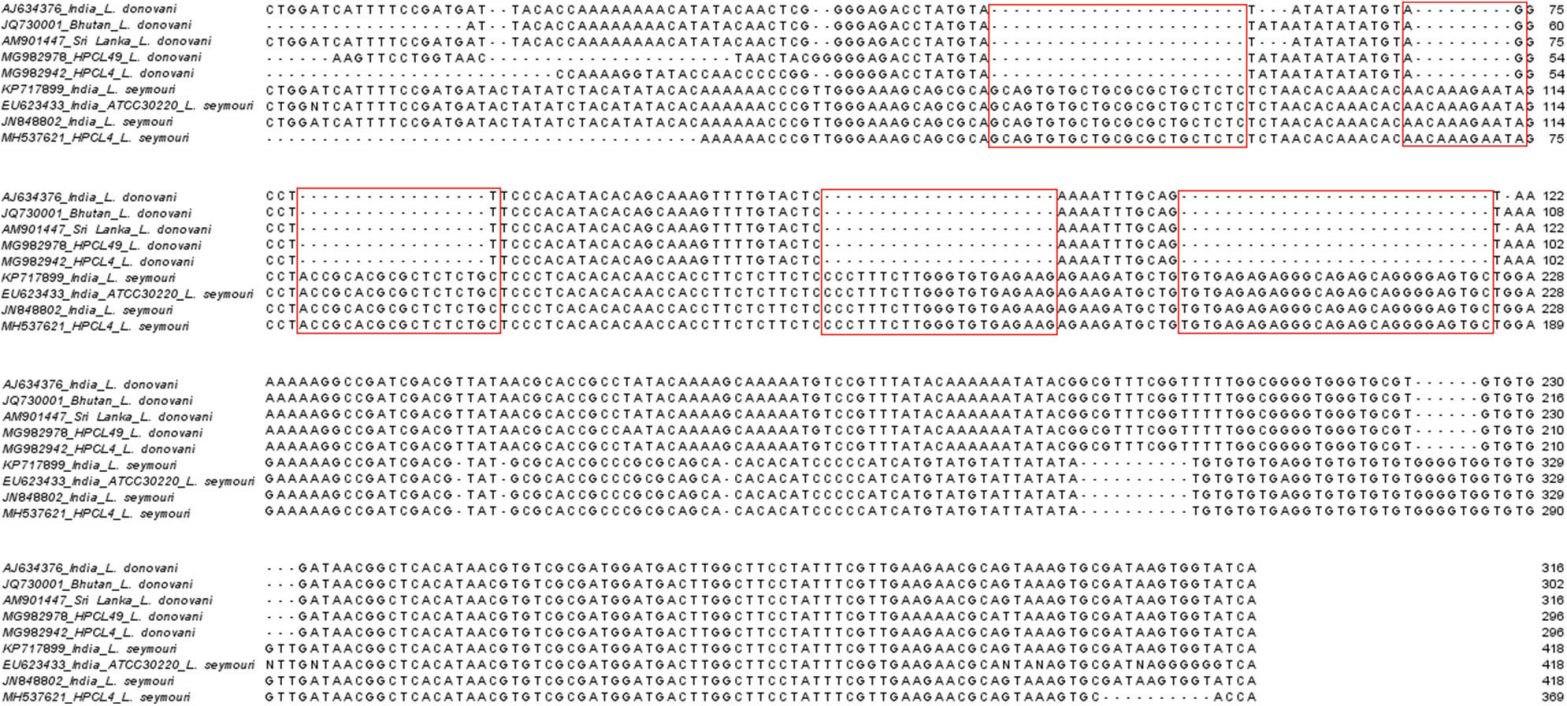
Multiple sequence alignment of ITS1 sequences representing *Leishmania* specific ~320 bp band (*MG982942_HPCL4_L. donovani* and *MG982978_HPCL49_L. donovani*) and *Leptomonas* specific ~400 bp band (*MH537621_HPCL4_L. seymouri*) along with standard *L. donovani* and *L. seymouri* specific ITS1 sequences retrieved from Genbank (*AJ634376_India_ L.‘ donovani, JQ730001_Bhutan _L. donovani, AM901447_Sri Lanka_ L. donovani, KP717899_ India_L. seymouri, EU623433_India_ ATCC30220_L. seymouri* and JN848802_India_*L. seymouri*). Sequences were aligned using the Jalview sequence alignment program. Differences in *L. donovani* and *L. seymouri* ITS1 sequences are highlighted in red.

ITS1 DNA region is highly conserved among different trypanosomatid parasite species (Dávila and Momen, 2000; Borghesan et al., 2013). Multiple sequence alignment of the sequences corresponding to the representative HP *L. donovani*, ~320 bp and HP *L. seymouri*, ~400 bp ITS1 sequences (MG982942, MG982978, MH537621) along with the ~320 bp ITS1 sequences of standard *L. donovani* isolates from India (AJ634376), Sri Lanka (AM901447), Bhutan (JQ730001) and ~400 bp ITS1 sequences of standard *L. seymouri* isolates from India (KP717899, EU623433, JN848802) exhibited distinct differences in ITS1 sequences specific to *L. donovani* and *L. seymouri*. Additional nucleotide patches unique to *L. seymouri* ITS1 sequence in comparison to *L. donovani* ITS1 region were apparent in the sequence alignment shown in Figure 3.

Detection of the ITS1 sequences corresponding to the two bands in clinical specimens clearly exhibited the presence of *L. seymouri* and *L. donovani* co-infection in CL patients from HP. We report *L. seymouri* as a co-infecting parasite in almost 38.5% (22/57) of the CL cases caused by *L. donovani*. This is the first-ever report of *L. seymouri* co-infection in CL patients, typed with unusual cutaneous manifestation by *L. donovani* with no visceral features, detailed in a recent communication from our laboratory (unpublished data).

## Discussion

Our study concludes another instance of *L. seymouri* as a *L. donovani* co-infectant in leishmaniasis patients from HP, a newer endemic region in India in line with earlier reports of *L. seymouri* co-infection in VL and PKDL patients from the northeastern VL zone (Srivastava et al., 2010; Ghosh et al., 2012; Singh et al., 2013). Importantly, the CL patients diagnosed in our study with *L. seymouri*, exhibit cutaneous manifestation caused by *L. donovani* (Sharma et al., 2005; and unpublished data). A possible role of *Leptomonas* co-infection in disease pathogenesis and phenotypic outcome with viscerotropic and/or dermotropic manifestation in VL vs. PKDL cases is not much explored. In addition to *L. seymouri* co-infection detected in cutaneous *L. donovani* disease in our study, the parasite has been reported from PKDL cases and HIV patients with a diffuse cutaneous phenotype (Dedet et al., 1995; Ghosh et al., 2012). Earlier notion of *Leptomonas* spp incapable of causing infection in vertebrates is getting blur with increasing evidence of its dixenous existence as a real co-infecting partner rather than a culture contaminant (Srivastava et al., 2010; Ghosh et al., 2012; Singh et al., 2013; Kraeva et al., 2015; Selvapandiyan et al., 2015). This is evident from the detection of *L. seymouri* in 22/57 clinical specimens directly processed for the molecular detection of the parasite with a rare chance of interim contamination.

*L. seymouri* is currently understood as an opportunistic parasite in immuno-compromised hosts such as HIV and Leishmaniasis cases (Kraeva et al., 2015; Selvapandiyan et al., 2015). Human cases of *Leptomonas* co-infection have been underestimated due to its morphological, antigenic and genomic similarity with *Leishmania* such that many of the DNA sequences specific to *L. seymouri* have been assigned to *L. donovani* (Nasereddin et al., 2008; Ghosh et al., 2012; Kraeva et al., 2015; Selvapandiyan et al., 2015). In this regard a rapid diagnostic method to detect and differentiate *Leishmania* and *Leptomonas* in clinical samples needs to be adopted for reliable experimentation and data inter-presentation along with specifically understanding the clinical correlates of *Leptomonas* co-infection (Ahuja et al., 2020). Interestingly, the genetic similarity of *Leptomonas* with *Leishmania* along with few nucleotide variations in ITS1 sequences among the two clusters retrieved in our study raises the possibility of the existence of genetic hybrids with heterogeneous *Leishmania* as well as *Leptomonas* genotypes albeit with limited evidence of natural *L. seymouri* infection in *P. argentipe* known for *L. donovani* transmission in India.

Importantly, *Leptomonas* like parasite has been demonstrated to naturally co-infect *P*. argentipes along with *L. donovani* in VL endemic region of Nepal (Bhattarai et al., 2009). Also, *L. seymouri* and *L. donovani* have been shown to co-persist in *P. argentipes* experimental infection (Kraeva et al., 2015). With the possibility of *P. longiductus* mediated *L. seymouri*-*L. donovani* co-infection in HP, natural co-infection of *P. longipalpis* by *Leptomonas* and *L. donovani* has been demonstrated in a focus of kala-azar (Deane and Deane, 1954; Sharma et al., 2009). Additionally, *P. longiductus* has been identified as one of the vector in Bhutan and China (World Health Organization, 2010; Yangzom et al., 2012). Thus, the identity of sandfly vector capable of co-transmitting *Leptomonas* and *Leishmania* parasite needs to be further ascertained in different regions with reports of *Leptomonas* co-infection in humans. The similarity in antigenicity of the two organisms further warranties a comparative immune-profiling of the VL and CL patients with and without *L. seymouri* co-infection relevant in immune response studies like deciphering *L. donovani* mediated immune-suppression that possibly makes infected patients vulnerable to *Leptomonas* co-infection and for vaccine development studies (Selvapandiyan et al., 2015).

In conclusion, the presence of *L. seymouri* with *L. donovani* in VL, PKDL and CL cases imply a real yet unappreciated phenomenon with the possible implication in disease outcome. We emphasize the emerging *L*. seymouri- *L. donovani* co-infected CL cases in the newer Sutluj river belt in HP in addition to co-infected VL/PKDL cases, reported in the northeast belt of India. The finding needs to be taken as a new challenge for the leishmaniasis surveillance and elimination program operational in India.

## Data Availability Statement

The datasets presented in this study can be found in online repositories. The names of the repository/repositories and accession number(s) can be found in the article/Supplementary Material.

## Ethics Statement

The studies involving human participants were reviewed and approved by Institutional Ethics Committee IGMC, Shimla, Himachal Pradesh, Approval no. HFW(MS)G-5(Ethics)/2014-10886 and Central University of Punjab, Approval no. CUPB/IEC/2016/034. Written informed consent to participate in this study was provided by the participants' legal guardian/next of kin.

## Author Contributions

LT collected the samples, performed the experiments, and analyzed the data. HK constructed the phylogenetic tree. AN provided the patient samples. AJ and MJ contributed in concept and design of study, data analysis, drafting of article, critical revision, and final approval of manuscript. All authors contributed to the article and approved the submitted version.

## Conflict of Interest

The authors declare that the research was conducted in the absence of any commercial or financial relationships that could be construed as a potential conflict of interest.
