# Supplementary figures and images for: Leptomonas seymouri Co-infection in Cutaneous Leishmaniasis Cases Caused by Leishmania donovani From Himachal Pradesh, India
Source: Front Cell Infect Microbiol. 2020 Jul 15;10:345. doi: 10.3389/fcimb.2020.00345 (PMC7373763; doi:10.3389/fcimb.2020.00345)

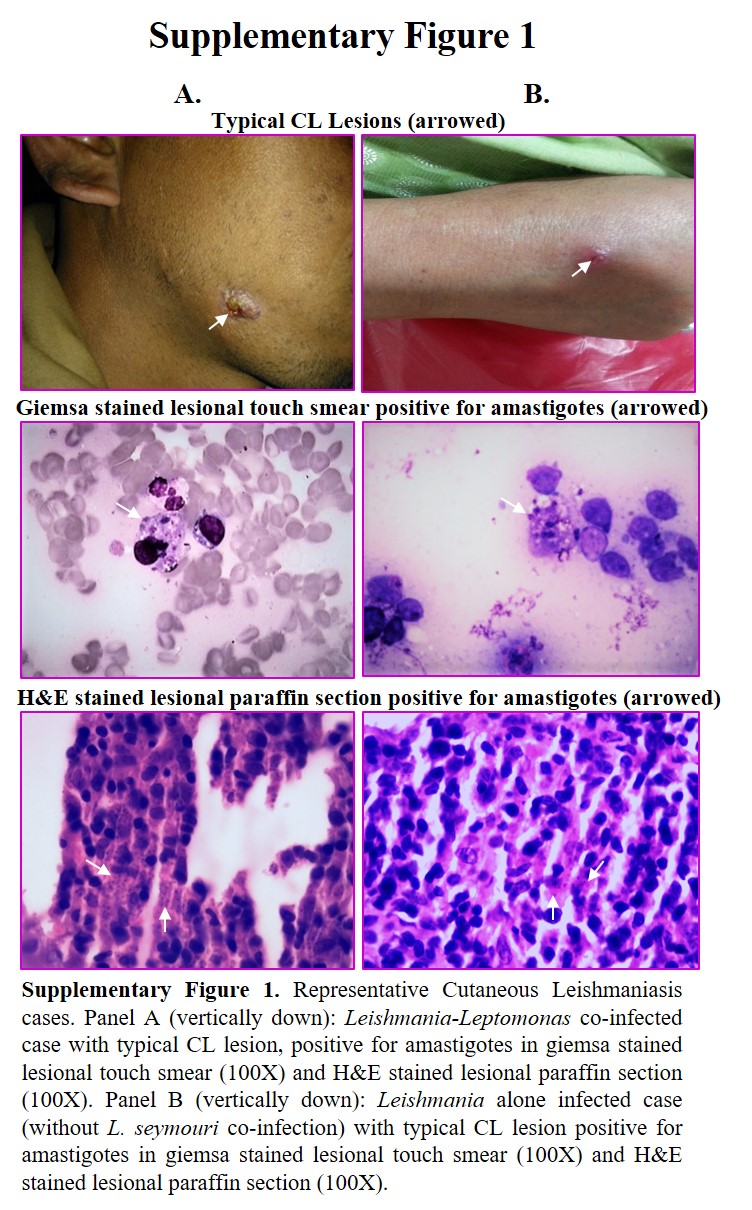

Supplement: Supplementary file 1 [file Image_1.JPEG]

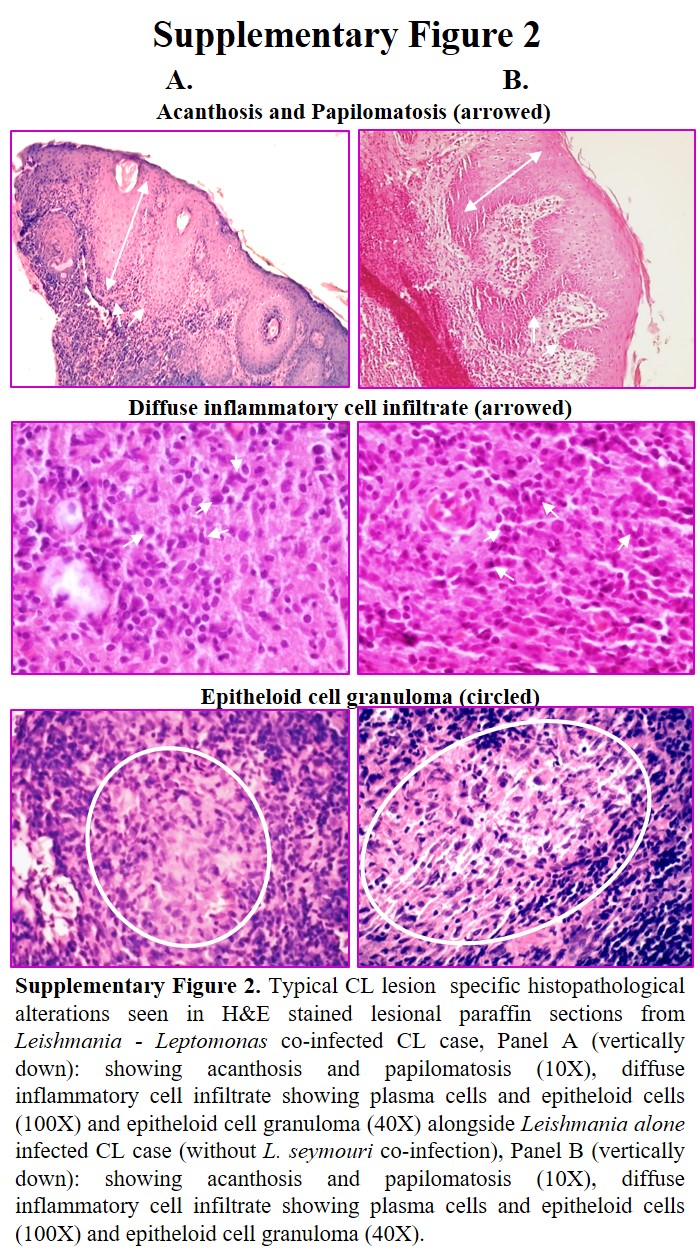

Supplement: Supplementary file 2 [file Image_2.JPEG]
