# Supplementary material for: Leptomonas seymouri Co-infection in Cutaneous Leishmaniasis Cases Caused by Leishmania donovani From Himachal Pradesh, India
Source: Front Cell Infect Microbiol. 2020 Jul 15;10:345. doi: 10.3389/fcimb.2020.00345 (PMC7373763; doi:10.3389/fcimb.2020.00345)
Supplement: Supplementary file 3 [file Table_1.DOCX]

**Supplementary Table 1**: Baseline characteristics and clinical findings of *Leishmania-Leptomonas* co-infected and *Leishmania* only (without *L. seymouri* co-infection) CL patients from Himachal Pradesh.

| **CL cases with *L. donovani*-*L. seymouri* co-infection** | | | | | | | | | |
| --- | --- | --- | --- | --- | --- | --- | --- | --- | --- |
| **No. of cases: 22** | **Age (years)** | | | **Size of Lesion (Range)** | **Number of Lesion (Range)** | **Duration till clinical diagnosis** | **Parasite detection**  **(Positive for Amastigotes)** | | |
| **M/ F** | **0-20** | **21-40** | **>40** |  |  |  |  | **Giemsa touch smears Ld +ve**  **= 6/17 (35%)** | **H&E biopsy sections Ld +ve = 7/17 (41%)** |
| **Male** = 13/ 22 | 4/ 14 (28.5%) | 8/ 14 (57.1%) | 2/ 14 (14.3%) | 0.3x0.3 cm to 3x2 cm | 1-4 | 1 month to 2.6 years | **+ve** | 3/ 11 (27.3%) | 5/ 11 (45.4%) |
|  |  |  |  |  |  |  | **-ve** | 7/ 11 (63.6%) | 6/ 11 (54.5%) |
|  |  |  |  |  |  |  | **Doubtful** | 1/ 11 (9.1%) |  |
|  |  |  |  |  |  |  | **Not Done** | 2/ 13 | 2/ 13 |
| **Female** = 9/ 22 | 2/ 8 (25%) | 4/ 8 (50%) | 2/ 8 (25%) | 0.7x0.7 cm to 3x2 cm | 1-2 | 1 month to 1.6 years | **+ve** | 3/ 6 (50%) | 2/ 6 (33.3%) |
|  |  |  |  |  |  |  | **-ve** | 3/ 6 (50%) | 4/ 6 (66.6%) |
|  |  |  |  |  |  |  | **Doubtful** | Nil | Nil |
|  |  |  |  |  |  |  | **Not Done** | 3/ 9 | 3/ 9 |
| **CL cases caused by *L. donovani* (without *L. seymouri* co-infection)** | | | | | | | | | |
| **No. of**  **Cases: 38**  **M/ F** | **Age (years)**  **0-20 21-40** **>40** | | | **Size of Lesion (Range)** | **Number of Lesion (Range)** | **Duration till clinical diagnosis** |  | **Giemsa touch smears Ld +ve = 17/26 (65.4%)** | **H&E biopsy sections Ld +ve = 12/30 (40%)** |
| **Male** = 16/ 38 | 6/ 16 (37.5%) | 6/ 16 (37.5%) | 4/ 16 (25%) | 2x2 cm to 5x5 cm | 1-4 | 25 days to 7 months | **+ve** | 6/ 9 (66.7%) | 5/ 11 (45.4%) |
|  |  |  |  |  |  |  | **-ve** | 2/ 9 (22.2%) | 7/ 11 (63.6%) |
|  |  |  |  |  |  |  | **Doubtful** | 1/ 9 (11.1%) |  |
|  |  |  |  |  |  |  | **Not Done** | 7/ 16 | 4/ 15 |
| **Female** = 22/ 38 | 6/ 22 (27.3%) | 8/ 22 (36.3%) | 8/ 22 (36.3%) | 0.7x0.7 cm to 3x3 cm | 1-3 | 1 month to 1 years | **+ve** | 9/ 17 (52.9%) | 7/ 19 (36.8%) |
|  |  |  |  |  |  |  | **-ve** | 5/ 17 (29.4%) | 12/ 19 (63.2%) |
|  |  |  |  |  |  |  | **Doubtful** | 3/ 17 (17.6%) |  |
|  |  |  |  |  |  |  | **Not Done** | 5/ 22 | 1/ 20 |
